# Adherence to Oral Hypoglycemic Agents in Hawaii

**Published:** 2005-03-15

**Authors:** Rachel Lee, Deborah A Taira

**Affiliations:** John A. Burns School of Medicine; John A. Burns School of Medicine, Honolulu, Hawaii, Hawaii Medical Service Association

## Abstract

**Introduction:**

Adherence to oral hypoglycemic agents is essential to reducing the poor health outcomes of populations at high risk for developing diabetes and its chronic complications. The goal of this study was to identify characteristics of patients in Hawaii least likely to adhere to oral hypoglycemic agents.

**Methods:**

This retrospective administrative data analysis included prescription refill claims for oral hypoglycemic agents from January 1, 1999, through June 30, 2003 (n = 20,685). Multivariate logistic regression analysis was used to examine the relationship between adherence and patient characteristics.

**Results:**

Adherence was found to be strongly associated with age and ethnicity. Relative to the age subset 55 to 64 years, adherence increased as age increased, reaching a peak at age 74 (odds ratio [OR] 1.1; 95% confidence interval [CI], 1.0–1.20). Past the age of 85, adherence declined (OR 0.90; 95% CI, 0.82–0.98). Relative to white patients, the odds ratio of adherence was highest for Japanese patients (OR 1.20; 95% CI, 1.0–1.30) and lowest for Filipino patients (OR 0.78; 95% CI, 0.68–0.90). Gender was not associated with adherence.

**Conclusion:**

Differences in adherence to oral hypoglycemic agents were found to be related to ethnicity and age. Adherence was found to be lowest in younger patients and Filipino patients. This is a significant finding considering that younger diabetic patients have been shown to have the poorest glycemic control and worst health outcomes. Although the literature on adherence to oral hypoglycemic agents and health outcomes in Filipino patients is limited, studies support an increased risk for developing diabetes in this group. This information can be used to target younger patients and Filipino patients to improve their adherence to oral hypoglycemic agents.

## Introduction

Patient adherence to a prescribed regimen of oral hypoglycemic agents to prevent diabetes is generally low and difficult to maintain, even in populations with adequate access to health care and drug coverage (1,2). This problem poses serious consequences for Asian-Pacific Islanders, who have a higher genetic predisposition than whites for developing diabetes and its chronic complications (3-6). The Asian-Pacific Islander population in Hawaii is large and heterogeneous, composed of individuals of Japanese, Chinese, Filipino, Korean, and Hawaiian ancestry. Comparing the different ethnic groups in Hawaii that compose the Asian-Pacific Islander category reveals significant health disparities among them. Diabetes has been found to be three to seven times more prevalent in Hawaiians and three to four times more prevalent in Filipinos and Japanese than whites (7). In addition, Hawaiians have the highest prevalence of diabetes reported for any Polynesian or part-Polynesian group, and mixed ancestry has not been shown to diminish the risk for type 2 diabetes (8,9). Native Hawaiians on average have less education and lower income and are more likely to live in rural areas with less access to medical services than other ethnic groups living in Hawaii. These aspects of the Native Hawaiian population further elevate its risk for developing diabetes and its chronic complications.

Evidence suggests that dietary and lifestyle changes place ethnic minorities at higher risk for developing diabetes. The Honolulu Heart Program, a study of 8006 Japanese men born from 1900 to 1919, correlated a westernized and sedentary lifestyle with an increased risk for developing diabetes. These studies found that Japanese men who retained a Japanese lifestyle and diet were less likely to develop diabetes than those Japanese men who followed a westernized lifestyle (10,11).

Different cultural beliefs and dietary practices among the various ethnic groups in Hawaii affect the outcome of diabetes and adherence to medications. The literature suggests that traditional cultural beliefs about family and the social support it provides when family members are ill play an important role in either encouraging or preventing individuals from seeking medical care for diabetes (12,13). Because minority populations and patients facing socioeconomic barriers to health care access have been shown to have the worst adherence to medications and poor glycemic control (14), determining the association between ethnicity and adherence to oral hypoglycemic agents among Japanese, Chinese, Filipino, Hawaiian, and white patients in Hawaii will help reveal the ethnic disparities that exist in Hawaii and identify those groups who most need to be targeted for intervention.

## Methods

### Study population

The patients in this study were enrollees in a large health care plan in Hawaii from January 1, 1999, to June 30, 2003, who met their algorithm for diabetes, had drug coverage, and filled at least one prescription for one of the following oral hypoglycemic agents: sulfonylurea, metformin, thiazolidinedione, and α-glucosidase inhibitors. To determine if a patient had diabetes, we used a diabetes algorithm that followed two decision paths. The first path looked for the presence of a diabetes diagnosis with specific treatment services; the second identified the presence of diabetes based on specific drugs and medical supplies, provided the member did not have gestational diabetes. The characteristics that we controlled for were age, gender, ethnicity, island of residence, morbidity level, year of treatment, and type of coverage (i.e., HMO, PPO, Medicare cost contract).

Morbidity level was assessed using the Johns Hopkins University's Ambulatory Care Groups (ACGs), derived from the mix of a member's diagnoses. In the original study, 51 combinations or ACGs resulted from applying multivariate techniques to maximize variance explained in use of services and ambulatory care charges (15). This method can be applied to large populations with numerous types of diagnoses to predict ambulatory care use and cost of care and to determine the burden of morbidity. High morbidity was defined as a morbidity level of four or five on a five-point scale.

### Sources of data

Patient age, gender, island of residence, morbidity level, comorbid conditions, and type of coverage were obtained from administrative data. This data did not include ethnicity of the patient. Self-reported ethnicity information was available for a portion of members in the study population from existing health-plan member satisfaction surveys. The satisfaction survey was a mailed questionnaire filled out by members to describe their experiences with health care services; on that survey, members were asked to check all that apply among 17 ethnic categories. These categories were chosen to be consistent with the Hawaii Department of Health's Hawaii Health Surveillance Program. In most cases, members who marked more than one race or ethnicity were categorized as "mixed." Any member who marked Hawaiian, however, was classified as Hawaiian regardless of what other categories he or she may have marked, because so few persons are of Hawaiian-only ancestry. We examined ethnic differences in oral adherence for the six main ethnic/racial groups in Hawaii: Japanese, Chinese, whites, Hawaiians, Filipinos, and Koreans. Members who marked other ethnicities, no ethnicity, or were mixed but non-Hawaiian were excluded from the analyses.

### Calculation of adherence

Treatment adherence for this study was calculated allowing small gaps between prescriptions. The maximum allowable gaps were based upon possession ratios, calculated as follows:

Possession ratio = days supplied for first prescription/(fill date of second prescription − fill date of first prescription)

A claim separated from a previous claim with a possession ratio of 0.8 or greater was considered adherent.

Days of adherence were calculated from the date of the first prescription until the end-of-supply of the last claim. For isolated claims, claims not within a possession ratio of 0.8 of other claims, the days of adherence were taken as the days of supply on the claim. The days of adherence for other claims were calculated as follows:

Days of adherence = date of last adherent prescription − date of first prescription + days of supply on last compliant prescription

The average days of adherence per year were calculated as the days of adherence divided by the days of enrollment in a drug plan since the first prescription date. Patients needed to have some drug enrollment and medical enrollment to be in the study. They did not need to be continuously enrolled for the entire study period. Days of adherence were looked at and adjusted by days of enrollment on an annual basis. The average number of days of enrollment per year was 354 days.

### Statistical analysis

Multivariate logistic regression analysis was used to examine the relationship between adherence and patient characteristics. All analyses were performed using Stata V7.0 statistical software (StataCorp, College Town, Tex).

## Results

Among the 39,536 patients on an oral hypoglycemic agent, 20,685 unique numbers met the inclusion criteria for the study (Table 1). The mean age (± SD) of the study sample was 63.7 years (± 12.01). Of the study sample, 54.4% were male and 45.6% were female. Among the study sample, 20.7% had high morbidity; 77.3% lived on Oahu, 12.1% on Hawaii, 5.8% on Kauai, 6.3% on Maui, 0.4% on Lanai, and 0.7% on Molokai. The distributions of oral hypoglycemic agents were as follows: sulfonylurea 44.0%, metformin 31.2%, α-glucosidase inhibitors 1.6%, and thiazolidinedione 23.2%. During the study period, 19.9% of patients also used insulin. Of these patients, 16.0% used sulfonylurea, 19.8% used metformin, 31.0% used α-glucosidase inhibitors, and 33.0% used thiazolidinedione. Patients on thiazolidinedione and α-glucosidase inhibitors have a higher percentage of insulin use.

Adherence differed according to drug class, age, ethnicity, and island of residence (Table 2). Overall adherence to oral hypoglycemic agents was low at 61.4%. Relative to sulfonylurea, the odds ratio of adherence was highest for metformin, followed by thiazolidinedione, and lowest for α-glucosidase inhibitors. Relative to the age subset 55 to 64 years, adherence increased with older patients, reaching a peak at age 74, then decreased for patients aged 85 and older. Age was controlled for because metformin and thiazolidinedione are less likely to be used among the elderly due to contraindications and adverse effects. Japanese patients were the most likely to be adherent, followed by Chinese, whites (referent group), Hawaiians, and Filipinos. Relative to Oahu, Lanai had the lowest odds ratio.

Compared with the fee-for-service members, HMO members were less likely to adhere to medication therapy. Adherence of members with Medicare coverage was similar to that of members in the fee-for-service plan. Members with the lowest morbidity level (i.e., fewest comorbid conditions) tended to be the least adherent to medication. Adherence tended to improve with increased morbidity but declined for those with the highest morbidity level. Gender and the year of treatment were not significantly associated with adherence.

## Discussion

Patient adherence to oral hypoglycemic agents is integral to reducing the health care costs and chronic complications of diabetes. Identifying which patients are at greatest risk for nonadherence to oral hypoglycemic agents is an important first step toward developing interventions that improve adherence.

Older patients had better adherence than younger patients to oral hypoglycemic agents, with adherence declining after age 85. A possible explanation for the better adherence among the older patients is that they are more knowledgeable and experienced with using the medications. However, with increasing age and burden of disease, adherence becomes more difficult to maintain over time. The finding of an association between age and adherence to oral hypoglycemic agents has helped to identify younger diabetic patients as a susceptible group requiring intervention to improve adherence. The diagnosis of diabetes made at an earlier age means these individuals will have an increased duration of exposure to hyperglycemia and as a consequence increased severity of microvascular complications (16). In addition, recent trends show an increased incidence of type 2 diabetes in younger individuals belonging to minority groups (17). This is reflected in current screening guidelines that recommend diabetes testing earlier (before the age of 45) for anyone belonging to a high-risk group, such as Asian-Pacific Islanders.

Ethnicity was also found to be a significant factor in adherence to oral hypoglycemic agents. Japanese patients had the highest adherence, and Filipino patients had the lowest adherence. Worrisome about this finding is that Filipinos in Hawaii have the highest prevalence of diabetes among the four largest Asian groups, which include Chinese, Japanese, Filipino and Korean (18). Among Filipino males living in Hawaii, diabetes is the third leading cause of death, and in Filipino females it is the second leading cause of death (19). More research is needed to understand the risk factors that contribute to Filipino susceptibility to developing diabetes and the reasons behind the low rates of adherence to oral hypoglycemic therapy in this group.

More studies also need to be done on patients living on the island of Lanai to find out the reasons behind the low rates of adherence. One possibility to consider is the rural setting of the island, which poses barriers to health care access for persons with diabetes.

This study did not examine the effects on adherence of combination drug therapy either with or without concomitant insulin use. Combination therapy has been shown to be associated with poorer rates of adherence than monotherapy (20-21). In clinical practice, sulfonylurea and metformin are usually prescribed by themselves as a first-line therapy because these two drugs are the only ones to demonstrate decreased vascular risk. Furthermore, although thiazolidinedione and α-glucosidase inhibitors are indicated for use as monotherapy, they are used more in combination therapy, thereby contributing to the decreased rates of adherence in these two medications. Insulin has been associated with lower adherence rates; however, it would be premature to make any conclusion at this point without further data on the use of combination therapy in this population.

Other limitations to our results are that the database for ethnicity is not complete, patients may have received free samples from their physicians, and the use of medical refill claims is an indirect method of measuring adherence. It is not known if patients are actually ingesting their medications; however, filling a prescription is a necessary first step for doing so. Another limitation was the lack of HbA1c levels, which would have provided a means for linking nonadherence with poor health outcomes in the population. To be able to link poor health outcomes with nonadherence in susceptible groups, such as younger patients and Filipino patients, would strengthen the conclusion that these groups are at high risk for the chronic complications of diabetes and need to be targeted for intervention. In addition, this study did not examine the effects of combination drug use on adherence.

Despite these limitations, prescription refill claims can be used as a tool to predict patient characteristics at highest risk for low adherence. Previous studies that have attempted to correlate demographic characteristics such as race, age, and gender with adherence have had inconsistent results (22-24). However, these studies used a small sample population. This study used a large sample population, and the trends demonstrated for race and age were consistent with other studies that used a large sample population (25-27). 

The findings in this study highlight the need to target younger patients, Filipino patients, and patients living in rural areas, such as the island of Lanai, for better glycemic control. Even with adequate access to health care services and drug coverage, there was low adherence to oral diabetic medications. This is a disturbing finding considering that the benefit of intensive glycemic control has been demonstrated by the U.K. Prospective Diabetes Study (28).

## Figures and Tables

**Table 1:** Characteristics of Patients with Diabetes Taking Oral Hypoglycemic Agents in Hawaii (n=20,685), January 1, 1999, to June 30, 2003^a^

**Mean age, years (±SD)**	63.7 (± 12.01)
**Male**	54.4
**Female**	45.6
**High morbidity^b^**	20.7

**Island of residence**	

Oahu	77.3
Hawaii	12.1
Kauai	5.8
Maui	6.3
Lanai	0.4
Molokai	0.7

**Oral hypoglycemic agent**	

Sulfonylurea	44.0
Metformin	31.2
α-glucosidase inhibitors	1.6
Thiazolidinedione	23.2

**Insulin use and oral hypoglycemic agent**	19.9

Sulfonlyurea	16.0
Metformin	19.8
α-glucosidase inhibitors	31.0
Thiazolidinedione	33.0

a Values are percentages unless otherwise indicated.

b High morbidity was defined as a morbidity level of four or five on a 5-point scale using the Johns Hopkins University’s Ambulatory Care Groups (ACGs).

**Table 2:** The Impact of Drug Class and Patient Characteristics on Adherence to Oral Hypoglycemic Agents in Hawaii, January 1, 1999, to June 30, 2003

**Characteristics**	**Adherence OR (95% CI)^a^**

**Oral hypoglycemic agent**	

Sulfonylurea	1.0 (ref)
Metformin	0.76 (0.73-0.78)
α-glucosidase inhibitors	0.42 (0.37-0.46)
Thiazolidinedione	0.48 (0.46-0.49)
**Insulin use**	0.82 (0.79-0.85)

**Sex**	

Male	1.0 (ref)
Female	0.97 (0.94-1.0)

**Age, years**	

1-18	0.20 (0.02-1.90)
19-24	0.30 (0.13-0.66)
25-34	0.47 (0.39-0.57)
35-44	0.72 (0.67-0.78)
45-54	0.86 (0.83-0.90)
55-64	1.0 (ref)
65-74	1.1 (1.10-1.20)
75-84	1.1 (1.0-1.10)
85+	0.90 (0.82-0.98)

**Race/ethnicity**	

White	1.0 (ref)
Japanese	1.20 (1.0-1.30)
Chinese	1.0 (0.89-1.20)
Filipino	0.78 (0.68-0.90)
Hawaiian	0.89 (0.77-1.0)
Other	0.87 (0.74-1.0)
Missing data	0.85 (0.76-0.96)

**Island of residence**	

Oahu	1.0 (ref)
Hawaii	1.10 (1.10-1.20)
Kauai	1.10 (1.0-1.20)
Maui	0.98 (0.92-1.0)
Lanai	0.79 (0.62-1.0)
Molokai	0.94 (0.79-1.10)

**Morbidity^b^**	

Morbidity 1	1.0 (ref)
Morbidity 2	1.30 (1.10-1.50)
Morbidity 3	1.40 (1.20-1.60)
Morbidity 4	1.40 (1.20-1.60)
Morbidity 5	1.30 (1.10-1.60)
Morbidity 6	1.10 (0.95-1.30)

**Year**	

1999	1.0 (ref)
2000	0.97 (0.93-1.0)
2001	1.10 (1.0-1.10)
2002	0.92 (0.87-0.97)

**Coverage**	

Fee-for-service	1.0 (ref)
HMO	0.89 (0.85-0.93)
Medicare	0.98 (0.87-1.10)

a OR = odds ratio; CI = confidence interval.

b Morbidity level was assessed using the Johns Hopkins University’s Ambulatory Care Groups.
